# Supplementary figures and images for: Neural stem cells traffic functional mitochondria via extracellular vesicles
Source: PLoS Biol. 2021 Apr 7;19(4):e3001166. doi: 10.1371/journal.pbio.3001166 (PMC8055036; doi:10.1371/journal.pbio.3001166)

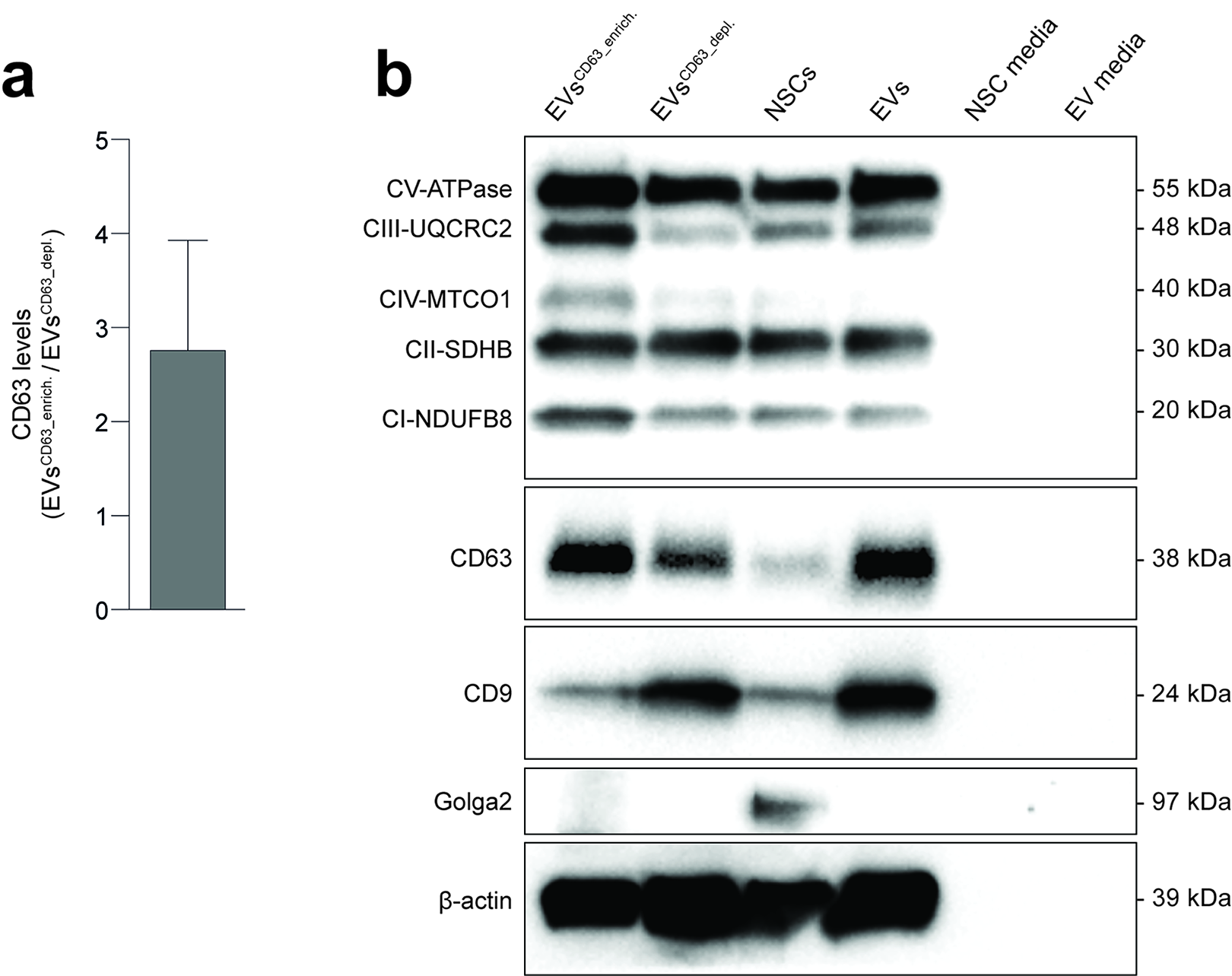

Supplement: S1 Fig — (a) Densitometry analysis of CD63 protein expression in EVsCD63_enrich. over EVsCD63_depl. (± SEM). N = 2 independent experiments. (Data available in S3 Data). (b) Protein expression analysis by WB of EVsCD63_enrich., EVsCD63_depl., NSCs, EVs, NSC media, and EV media. Mitochondrial complexes proteins (CV-ATPase, CII-SDHB, CIV-MTCO1, CIII-UQCRC2, and CI-NDUFB8), EV enriched proteins (CD63 and CD9), and negative (Golga2) markers are shown, as well as β-actin. The same amount of input material was loaded into each well. EV, extracellular vesicle; NSC, neural stem cell; WB, western blot. (TIF) [file pbio.3001166.s001.tif]

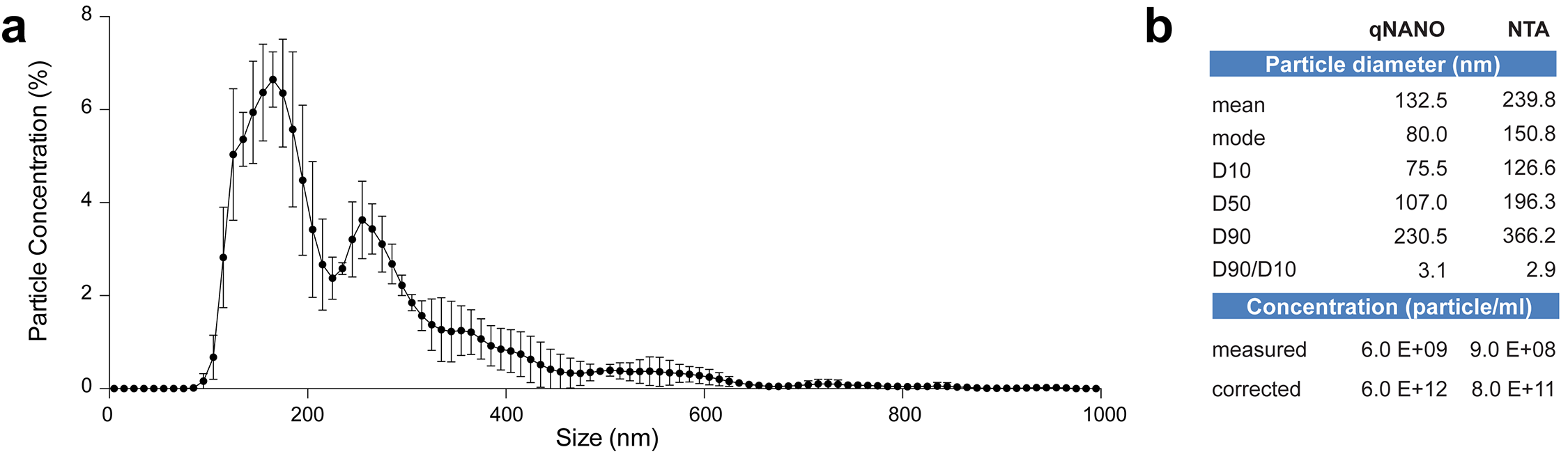

Supplement: S2 Fig — (a) Representative particle size distribution analysis of EVs by NTA, showing bimodal distribution of particle size in the exosome size range (30–150 nm) and in the submicron region (20–1,000 nm). Data are mean values (± SD). (Data available in S3 Data). (b) Comparison of EVs particle diameter (nm) and particle concentration (particle/ml) measurements using TRPS qNANO and NTA analysis. Data are expressed as mean values of N = 3 independent experiments. EV, extracellular vesicle; NSC, neural stem cell; NTA, nanoparticle tracking analysis; TRPS, tunable resistive pulse sensing. (TIF) [file pbio.3001166.s002.tif]

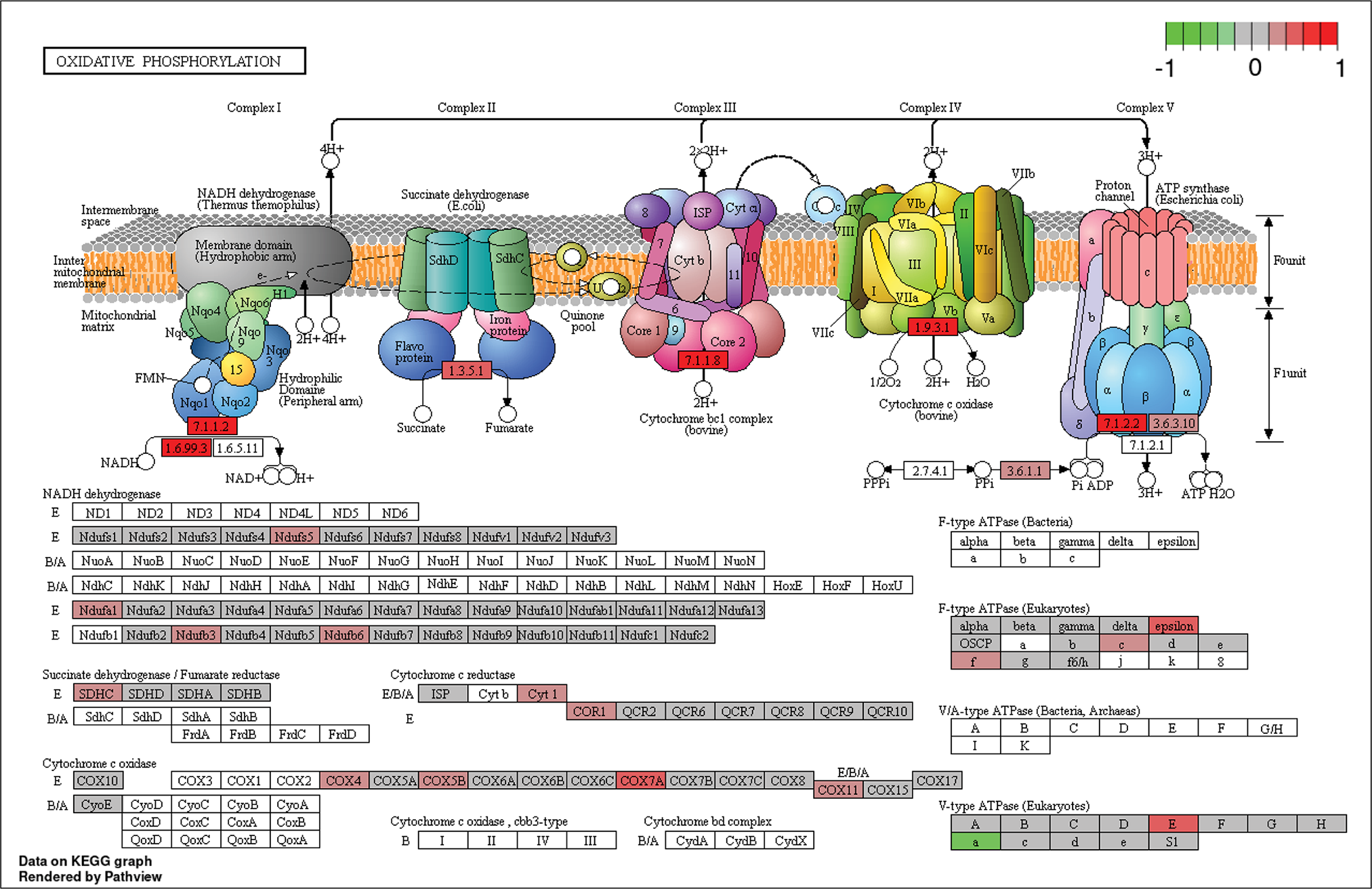

Supplement: S3 Fig — Pathview diagram showing the significantly enriched OXPHOS KEGG pathway. The colour scale represents the log2 fold change of each gene in EV-treated vs. untreated MφLPS as measured by microarray analysis. (Data available on ArrayExpress, identifier E-MTAB-8250). EV, extracellular vesicle; KEGG, Kyoto Encyclopedia of Genes and Genomes; LPS, lipopolysaccharide; OXPHOS, oxidative phosphorylation. (TIF) [file pbio.3001166.s003.tif]

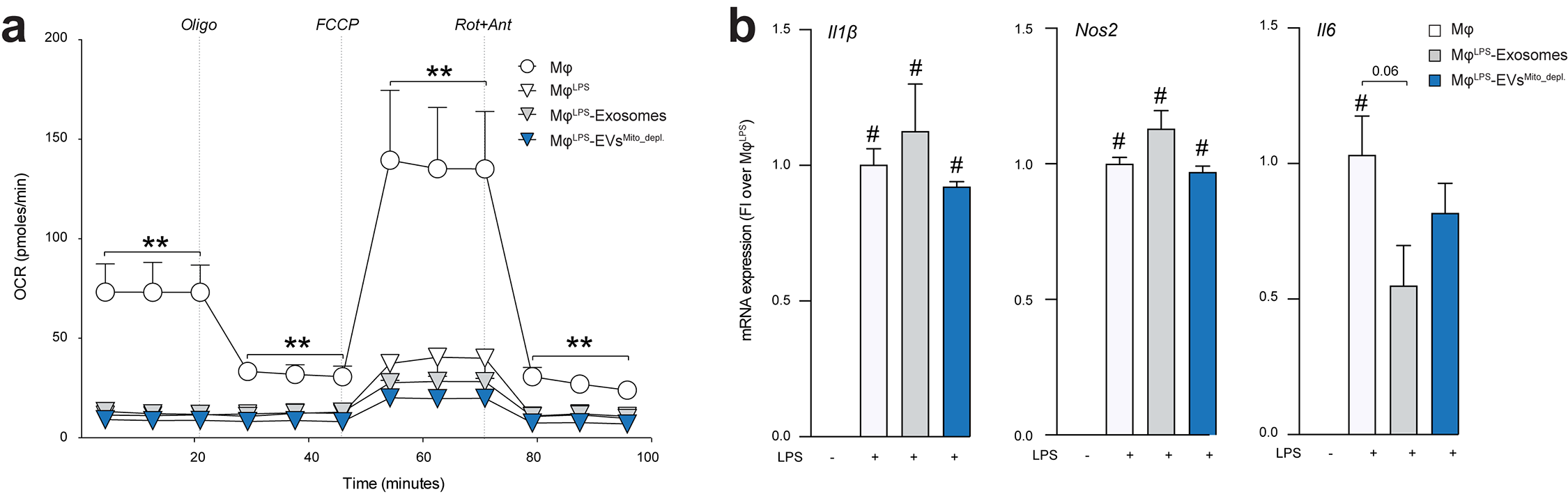

Supplement: S4 Fig — (a) XF assay of the OCR during a mitochondrial stress protocol of MφLPS at 6 hours from treatment with exosomes or EVsMito_depl vs. MφLPS. Unstimulated Mφ were used as controls. Data are mean values (± SEM). **p < 0.01 vs. MφLPS. N = 5 technical replicates per condition. (Data available in S3 Data). (b) Expression levels (qRT-PCR) of pro-inflammatory genes (Il1β, Nos2, and Il6) in MφLPS at 6 hours from treatment with exosomes or EVsMito_depl. Data are mean FI over MφLPS (± SEM). N = 2 biological replicates from N = 2 independent experiments. #p < 0.05 vs. unstimulated Mφ. (Data available in S3 Data). EV, extracellular vesicle; FI, fold induction; LPS, lipopolysaccharide; OCR, oxygen consumption rate; qRT-PCR, quantitative real-time polymerase chain reaction; XF, extracellular flux. (TIF) [file pbio.3001166.s004.tif]
