# Supplementary material for: Neural stem cells traffic functional mitochondria via extracellular vesicles
Source: PLoS Biol. 2021 Apr 7;19(4):e3001166. doi: 10.1371/journal.pbio.3001166 (PMC8055036; doi:10.1371/journal.pbio.3001166)
Supplement: Attachment — Submitted filename: PBIOLOGY-D-20-00273R1_Point by point reply.pdf [file pbio.3001166.s009.pdf]

Reviewer #1

1. *In the manuscript by Peruzzotti-Jametti et al., the authors investigate intercell mitochondrial trafficking. The authors find that NSCs shed mitochondria/mitochondrial fragments via extracellular vesicles and these vesicles can be taken up by adjacent cells. The authors show that uptake of these EVs can improve mitochondrial function in Rho-zero cells as well as inflammatory macrophages. Finally, the authors show that in vivo transplant of NSCs, or injection of EVs can improve symptoms in a mouse model of MS. This is a very intriguing manuscript that implies functional mitochondria can be transported between cells to influence their metabolism/behaviour. This has the potential to have significant implications for not just biology but also therapeutic approaches. However, I feel that the authors have not yet demonstrated that all the effects seen are due to mitochondrial transport and not some other element of EVs - and it may well take significant work to rule this out. Without solid data for mito-EVs, and not EVs in general, the manuscript too correlative.*

R1. We thank this reviewer for acknowledging the novelty of our findings and recognizing their potential therapeutic value. We agree with him/her that additional controls were indispensable to support a predominant role for mitochondria in our EV preparations as a major determinant of the observed effects on target cells. Furthermore, to simplify nomenclature and avoid confusion related to the term "Mito-EVs", we now use only the term "EVs" when referring to vesicle preparation containing mitochondria. Thanks to this reviewer's guidance, we now provide an extensively revised version of the manuscript, which we hope addresses in full the reviewer's concerns.

2. *The authors' data are clear in that NSCs can "shed" mitochondria, but what percentage of EVs actually contain mitochondria/mitochondrial fragments?*

R2. This is a very relevant question. To address this point, we now provide a quantitative assessment of the fraction of EVs that contain 'shed' mitochondria via state-of-the-art Nano Flow Cytometry (NanoFCM) measurements. Using fluorescent probes for a widely accepted EV marker (i.e. CD63) and a dye that specifically accumulates in functioning mitochondria (i.e. Mitotracker red), we have now determined that 33.5% of the particles in the EV preparation were positive for Mitotracker red. This new data is in line with our previous morphological TEM data (27.7% of the total particles). Furthermore, of all the mitochondria in the EV preparations, 77.6% were also expressing the EV marker CD63, thus suggesting co-localisation. These new data are reported in the **revised Figure 4b**, and in the text (changes are in red).

3. *The mito-EV preparations that the authors use also contain EVs that do not have mitochondria associated with them, so how are they certain that the effects seen are due to mitochondrial transfer itself? The authors need a control where they can generate the EVs without mitochondria and show these have no effects. I'm sure this is not a trivial matter and relates to the actual mechanism of how mitochondria get into these vesicles in the first place. The autophagy machinery has been linked to this - so perhaps inhibiting the initial stages of autophagy could produce EVs without mitochondria? Alternatively, could cells be depleted of mitochondria before collecting EVs (PMID: 28005069)?*

R3. This is another very relevant comment, which we addressed applying two complementary approaches.
First, we have applied the method established by Wang et al., 2017 (which includes a 0.22 μm filtration step) to remove intact mitochondria from the EV preparation, as also suggested by **Reviewer #3**. This method led to a total depletion of functional mitochondria from EVs, as shown by the NanoFCM data (**revised Figure 4b**) and by the loss of active mitochondrial potential (**revised Figure 4e**). When we used these mitochondria depleted EVs (named $EVs^{Mito\_depl.}$) to either treat LPS-treated (classically-activated) macrophages *in vitro* or to treat

EAE mice *in vivo*, we saw no functional effects (see **new Figure S4** and **revised Figure 9b**), which implies that the mitochondrial component of EVs mediates most of their functional effects on target cells.
Second, given the predominant association between the EV marker CD63 and mitochondria in our EV preparation (**revised Figure 4b** and **new FigureS1**), we also used an immune-mediated isolation protocol to deplete CD63 positive particles from our EV preparations. Also these EVs$^{CD63_depl.}$ failed to improve clinical deficits when injected ICV into mice with EAE (**revised Figure 9b**). Therefore, the CD63^{+} fraction of EVs (which is enriched in mitochondria) is indispensable for their therapeutic effect *in vivo*. These findings are discussed in the text in red.

4. *The authors use dsRed-mito to localize mitochondria in recipient cells. Are the authors sure that this red signal is still mitochondrial? For example, the process of mitophagy could lead to delivery of dsRed tagged mitochondria to MVBs/late endosomes/lysosomes whereby partial digestion could occur to release the label from the mitochondria. If the MVBs/late endosomes/lysosomes then fuse with the plasma membrane, this could lead to release of dsRed unattached to mitochondria - which could then be taken up by other cells.*

R4. Fusion of the dsRed fluorescent marker to a genomic sequence targeting mitochondria has previously been reported to reliably and specifically label mitochondria, as shown by confocal microscopy[1,2]. Although spill over is possible, as with many fluorescent dyes, the mitochondrial target sequence guarantees that the dsRed protein is accumulated only in those mitochondria conserving an intact membrane, greatly reducing the specificity of this tag[3]. Besides discussing these previous findings in the main body of the paper, we are now also providing new correlative light electron microscopy (CLEM) images showing co-localisation between the MitoDsRed protein and ultrastructurally defined mitochondria in macrophages treated with MitoDsRed^{+} EVs, as reported in the **revised Figure 6g**. These findings support the notion that the MitoDsRed signal remains associated with intact (and ostensibly functional) mitochondria after transfer to recipient cells. Changes in the text are in red.

5. *Related to this, in Figure 6 (and others) the authors show dsRed structures in the cell at various locations - including at the mitochondria. The authors imply that these dsRed-mitochondria become "integrated in the mitochondrial network", but how do they know this? The authors demonstrate that endocytosis is needed to uptake mito-EVs, thus they will be surrounded by at least one limiting membrane (maybe more if the EV membrane is taken into account) - how then do the mitochondria escape these membranes to become integrated into the network? My feeling is that these red structures could be endosomal in nature. Indeed, endosomes and lysosomes make extensive contacts with mitochondria without ever directly joining up with them. The best way that I can think of to address these two points would be to carry out CLEM, which would show directly that the red dots are indeed mitochondria and that they integrate into the mitochondrial network and are not simply endocytic cargo.*

R5. We thank the reviewer for this suggestion, which we have received and implemented in full. To address this key point, we have adopted two complementary techniques.
First, as suggested by the reviewer, we now provide representative CLEM images depicting exogenous MitoDsRed^{+} mitochondria integrated in the host mitochondrial network of macrophages treated with EVs, in the **revised Figure 6g**.
Second, we used a split self-associating fluorescent protein (FP) system that identifies fusion events between EVs (carrying the fragment 1-10 of the sfCherry2 FP) and the endogenous mitochondrial network of macrophages (expressing the fragment 11 of the sfCherry2 FP fused with the mitochondrial TOMM20 protein). Images reported in the **revised Figure 6f** show the presence of the fusion sfCherry2 protein in macrophages treated with EVs, thus confirming the fusion with the endogenous mitochondrial network. We hope that these two approaches satisfy in full the reviewer's concerns. Changes in the text are reported in red.

6. *I notice that for some of the imaging data, only the Imaris renderings are shown (e.g. Fig 6d). While these are very nice images, setting up the masking for the 3D mapping can be somewhat subjective, hence I think it is important to also include the original micrographs too. Related, for Figure 8a it looks like the three of the mitochondrial images are normal, while the M-LPS one has been processed through Imaris - they should all be the same.*

R6. We thank the reviewer for these suggestions, and we agree that 3D mapping can be somewhat subjective. We are now providing new, more detailed, original micrographs (with z-stack and 3D mapping) showing the co-localisation of MitoDsRed particles with the host mitochondrial network in a **revised Figure 6e**. As further suggested, we have also replaced the representative image of the $M\varphi^{LPS}$ in **Figure 8a**.

Reviewer #2

7. *The authors provide a well written, exciting paper that adds to the understanding of the transfer mechanism and functional relevance of extracellular vesicles/ectosomes-transmitted mitochondria from neural stem cells. The authors have presented a significant amount of data to unpick the role of EVs in shuttling mitochondria, covering the functionality in vitro and in vivo. Moreover, they have addressed a range of aspects of this phenomena, from the molecular functions of the mitochondria to identifying the recipient cells.*

R7. We thank this reviewer for acknowledging the novelty of our findings. We are now providing an extensively revised manuscript that integrates all the reviewer's suggestions. In addition, to simplify nomenclature and avoid confusion related to the term "Mito-EVs", we now use only the term "EVs" when referring to vesicle preparation containing mitochondria. We believe that these changes have significantly improved the quality of our approach and provided further data on key controls and experiments.

8. *The authors have addressed some of the limitations of their techniques and methods in their discussion in detail. My major concern with this paper, however, has not been addressed, and that is the contribution of EV's present in the cell culture media. The NSCs are cultured in Stem Cell Technologies Neural basal media, with additional standard Proliferation Supplements. This contains human plasma, which in itself will be a source of EVs. Later experiments use mitochondrial Mito-DsRed fluorescent reporter to label NSC derived mitochondria, providing clear evidence that mitochondria are transferred from the NSC to a recipient cell. However, there is still the possibility of contaminating plasma-derived EVs in the in vivo experiments, for example, that could be contributing to the disease ameliorating effects seen. At the very least, the authors could use various analysis techniques to investigate the EV content of their culture media without NSC, and then confirm by Western blot that the profile of EV harvested from NSC grown in normal and EV-depleted culture media is the same. I appreciate that the NSC may not differentiate or grow well in EV-depleted media and that to repeat everything in EV-depleted media is not feasible. Still, it is essential to quantify the amount and effect of media-contributed EVs in this system. It would only require that the NSCs are grown in EV-depleted media for long enough to harvest NSC derived EVs.*

R8. We thank the reviewer for this criticism, which has been well taken and addressed in full. We are now providing further clarification on the composition of media used to expand NSCs (NSC media) and the media used to harvest EVs from NSCs (EV media) in the methods section. In addition, we are providing a fully dedicated **new Figure S1b** showing the absence

of either EV or mitochondrial markers from either NSC media or EV media by western blot. Changes in the text are reported in red.

9. *Furthermore, it is unclear to me how mitochondria-containing EVs are taken up in the receiving cells. Do the authors believe that the EV membrane fuses with the plasma membrane and the mitochondria are released in the cytoplasm? Are EVs endocytosed and routed to early endosomes? It is essential to address this point as it is important for the understanding of mitochondria function in the recipient cells. For example, the observations from the cryo-EM analysis could allow the authors to conclude how EVs/mitochondria are taken up.*

R9. We thank the reviewer for these useful comments. Our lab has previously addressed the issue of EV intake into target cells (i.e. NIH 3T3 cell line) by flow cytometry, confocal, and stimulated emission depletion (STED) super-resolution microscopy[4]. We showed that EVs rapidly adhere to, and are incorporated into, target cells via the plasma membrane, with no significant association between EVs and the lysosomal-associated membrane protein LAMP-1, thus excluding direct or immediate degradation in target cells[4]. In the present manuscript, we confirm these findings by using professional phagocytes (i.e. bone marrow-derived macrophages) as target cells and demonstrate that endocytosis is the prevalent method of EV incorporation into target cells.
However, we agree with this reviewer - as well as with **Reviewer #1** - that additional data was needed to determine the fate of our EVs in macrophages. As such, we have managed to perform new additional experiments using two complementary techniques.
First, we applied correlative light and electron microscopy (CLEM) as in **R5**. In addition, we we have also carried out a split self-associating fluorescent proteins (FPs) experiment to detect EVs to macrophages' mitochondria contacts, as in **R5**.
Altogether these data clarify the intracellular fate of the EVs after endocytosis, suggesting trafficking, fusion, and integration within the host mitochondria network. The previously published evidence and these new data are now included in the text (changes are reported in red).

10. *Based on a recent review from Raghu Kalluri and Valerie S. LeBleu in Science, 2020, microvesicles and apoptotic bodies belong to the so-called ectosomes. The authors may use the revised definition of extracellular vesicles in their work.*

R10. We thank the reviewer for this comment. We have further elaborated on the recent changes of EVs nomenclature in the introduction, as suggested. Changes in the text are reported in red.

11. *Check the formatting of references throughout; numbering is not sequential.*

R11. The format of the references was a consequence of our submission to bioRxiv and subsequent direct transfer of the manuscript to PLOS Biology. We apologise for this. We have now updated the references' style, as suggested.

12. *In Fig 2a, some proteins such as CD9, beta-actin, etc., from the different EV preparation methods show different sizes. The authors need to clarify why this is.*

R12. Samples isolated by using the commercially available kits (e.g. Qiagen) have been obtained by following the manufacturer's instructions, which included resuspension of the sample in proprietary buffers as a final step prior to sample preparation for SDS-PAGE. While all the samples have been processed using the same protocol for SDS-PAGE and Western

blot, as per the methods section, it is likely that such commercial buffers might contain interfering substances that affected protein migration on SDS-PAGE (such as salt, detergent/denaturants, or organic solvents). Unfortunately, due to the proprietary nature of buffer composition, we are not able to exactly identify the aforesaid substance. We also contacted the technical service, and they were able to confirm such a possibility, but were not willing to reveal any further details. Nevertheless, we are confident that our samples were not compromised, as no smeared signal was observed, and a single strong band was observed for each antibody, excluding non-specific binding. We have now clarified the possible artefacts introduced by the precipitation-based isolation protocols in the figure legend. Changes in the text are reported in red.

13. *Check the formatting of Fig. 2. The citations at the top are cut off.*

R13. Thank you for noticing this. We have corrected Figure 2.

14. *At the bottom of page 22, relating to Fig 7, the authors should discuss the finding that cytochalasin D treatment also reduces the uptake of MitoDsRed EVs, not just D/P. The effect of cytochalasin D is, in fact, greater than D/P, but is not mentioned.*

R14. We thank the reviewer for this additional comment. This point has now been cogently addressed in the revised manuscript (results sections). Changes in the text are reported in red.

Reviewer #3

15. *The manuscript by Peruzzotti-Jametti et al reports the observation that NSCs ship via Extracellular Vesicles (EVs) intact mitochondria (mito), which integrate into the mitochondrial network of LPS stimulated macrophages, restoring damaged respiration and mitigating the proinflammatory phenotype. The data suggest that EV-mito transfer from NSCs to macrophages may play an immunomodulatory role under neuroinflammatory conditions and has the potential ability to ameliorate disease conditions such as those observed in EAE, the main mouse model of multiple sclerosis. In my view, the study reveals novel aspects regarding the immunomodulatory functions of NSCs, which are of potential therapeutic relevance. The study comprises detailed molecular and morphological EV-characterization, in vitro functional analyses as well as promising in vivo data. However, while the transfer of functional mitochondria at least in vitro is convincingly demonstrated, I'm not entirely convinced that mitos indeed take their route via EVs (could be shuttled via another transcellular route). The study shows that EV-fractions contain free mitos and the presented experiments do not allow to discriminate the functional impact of free and EV-associated mitos. Thus, the route of delivery is not completely solved and either should be further addressed or better discussed.*

R15. We thank this reviewer for acknowledging the value of our study and the novelty of our findings. We are now providing further data to reply to this reviewer's concerns, which are included in the present version of the manuscript and highlight the functional role of intact mitochondria in the EV preparations, as well as the therapeutic role of specific EV fractions (i.e. CD63 enriched and depleted). In addition, to simplify nomenclature and avoid confusion related to the term "Mito-EVs", we now use only the term "EVs" when referring to vesicle preparation containing mitochondria. Finally, we have better discussed the limitations of our approach and the possible impact of both free or EV-associated mitochondria in the discussion (changes are in red). We hope that these changes will now satisfy this reviewer's concerns.

16. *Neither biochemical, morphological or functional data provide a final proof of EV-mediated transfer (see specific points below). This is partly due to technical limitations difficult to address. However, all functional experiments were performed with "crude" EV-fractions also containing free mitos. In the EAE experiment, multiple factors contained in the highly complex mito-EV-fraction may contribute to the observed amelioration. To provide further evidence of EV-mediated transfer a method involving EV-depletion should be employed (immune-mediated depletion or pharmacological/genetic manipulation of NSC to interfere with EV-release).*

R16. This is an extremely well taken point and, as the reviewer pointed out, difficult to fully address due to many technical limitations.
Nonetheless, we accepted in full this criticism, and we followed the reviewer's suggestion to employ additional methods that involve EV-depletion, both *immune mediated* and *physical.*
As requested, we are now providing additional *in vivo* EAE data on mice treated with EVs obtained via (1) a method of EV-depletion based on a negative selection with CD63 immunobeads and (2) a method to deplete mitochondria from EVs based on an additional filtration step (Wang et al 2017).
Our behavioural analysis shows that both of these preparations failed to ameliorate EAE-associated behavioural deficits in mice, when injected intracerebroventricularly at peak of disease. These new data, which are now reported in the **revised Figure 9b,** suggest that the CD63 enriched fraction of EVs is necessary for their therapeutic effect *in vivo*, thus providing a better understanding of the high complexity of EVs released by NSCs. Changes in the text are reported in red.

17. *Alternative EV isolation protocols are used to support the data obtained with EVs isolated by differential centrifugation (Fig. 2). However, it should be clear that these are precipitation-based kits, which are known to co-isolate many other components (please add more information about the commercial EV isolation procedures at least to methods section).*

R17. We thank the reviewer for her comment. We have revised both the main text and the methods section to further discuss the use (and limitations) of precipitation-based kits. Changes in the text are reported in red.

18. *With regard to EV purity, a better alternative would be immuno-bead isolation (such as CD63 beads, which are commercially available). The Wang et al., 2017 method includes a 0.22 μm filtration step that should actually remove intact mitochondria, which typically range between 0.5 and 1 μm. The signals associated with this fraction thus likely reflect mitochondrial fragments contaminating the isolated EVs/exosomes.*

R18. As the reviewer suggested, we have further characterised the EV preparation using CD63 immuno-bead isolation. Our new data, reported in the **new Figure S1**, show a predominant expression of mitochondrial proteins in the CD63 enriched fraction of EVs. This finding fits well with additional NanoFCM data (provided in the **revised Figure 4b**), which show co-expression of CD63 with mitochondrial dyes and further support our *in vivo* findings suggesting that the CD63 fraction of EVs is necessary for a significant therapeutic effect in EAE (**revised Figure 9b**).
We have also better discussed the findings obtained using the method of Wang et al., 2017 in the text, and we have indeed used this method to physically deplete intact functional mitochondria from the EV preparation, as shown by our NanoFCM (**revised Figure 4b**) and JC1 Data (**revised Figure 4e**). Changes in the text are reported in red.

19. *Sucrose density gradient isolation was used to enrich for exosomes as a specific subtype of EVs and is assumed to provide EVs of higher purity due to the removal of non-EV components in the density gradient. Which criteria served to qualify fractions 6-9 as exosome containing fractions (please specify)? Indeed, Tsg101 WB shown in Fig. 3c indicates that fractions 5+6 rather qualify as exosome fractions, while fractions 7+8 may reflect high density non-exosomal material co-isolating with exosomes during differential centrifugation (Jeppesen et al, 2019).*

R19. Fractions 6-9 were qualified as containing exosomes based on previous published protocols[4,5]. While Tsg101 has classically been used as an exosome marker, Jeppesen et al. 2019 makes the case for this protein as a potentially better marker of Arrestin-domain-containing protein 1 (ARRDC1)-mediated microvesicles (ARMMs). Given the ever-evolving characterisation criteria of exosomes, we have employed the previously established density gradient protocol to maintain consistency with prior studies. We have now explained the parameters of density in the text. Changes in the text are reported in red.

20. *The mtDNA (Fig. 3d) spreads across the whole gradient (similar to actin) and it is difficult to see any enrichment in specific fractions rather looking like a non-specific association with all fractions. DNAse digestion could solve that question: In case of EV-association (or presence of intact free mitochondria) the DNA should be DNAse-resistant.*

R20. This is a crucial point raised by the reviewer. Indeed, all the EV preparations in Figure 1e and 3e were treated with DNAse I. We made sure this is explicitly reported in the main text (besides the methods section), as it strengthens our hypothesis of free and EV-associated intact mitochondria. Changes in the text are reported in red.

21. *When screening for typical exosomal markers in the proteomics results (Table S1), these are enriched in the crude EV fraction, but rather depleted in what is considered as exosome fraction. On the other hand, the proteins enriched in the exosome fraction (compared to crude EVs) are largely mitochondrial proteins. Page 20 (last paragraph) refers to proteins selectively depleted in exosomes versus EVs. What are these proteins and is this indeed an argument of exosome-enrichment in the collected fractions? The paper contains valuable proteomic information that could be better presented to the reader. It would be extremely helpful for the reader if the authors could add some sorted data including a list of proteins enriched in EVs/NSCs, Exo/NSCs, and EVs/Exo (please define selection criteria). Furthermore, Venn diagrams showing overlaps between NSC, EVs, and Exo fractions would be informative and complement the volcano plots.*

R21. We thank the reviewer for her comments on our proteomic data. We appreciate this paper provides valuable information, which we have now reorganised in a **revised Table S1**. In the "complete proteomic data" worksheet, we have highlighted q values <0.05 and data is now sortable allowing for easy identification of proteins that are significantly enriched *vs* NSCs. In addition, we have added a "gene search and plots" worksheet, which allows the reader to query data for individual proteins. As further requested, we are now also including a Venn diagram comparing proteins enriched in EVs and exosomes *vs* NSCs in a **revised Figure 3a**. Changes in the text are reported in red.

22. *Fig. 4: EM images are not convincing. In Fig. 4a, I cannot identify EV-encapsulated mitochondria marked by the arrowhead. Also, it does not fit with the mean diameter reported in the graph shown below and the text (page 22). Can it be replaced by a more representative image? Is the mitochondrion depicted in Fig. 4b encapsulated in an EV? In fact, there should be three membranes. Furthermore, TOMM20 is a translocase sitting in the outer membrane.*

Most gold particles actually indicate inner membrane or even somatic localization of the detected antigen.

R22. We have reviewed our approach to the cryo-TEM to increase the labelling of mitochondria encapsulated in EVs. To achieve this aim, we have included saponin in the antibody staining solution to allow better permeability of the gold-conjugated antibodies. We are now providing a more convincing representative image of a TOMM20$^+$ mitochondria encapsulated in CD63$^+$ EVs showing three distinct membranes, as reported in **revised Figure 4d**. We have also further clarified in the methods that our cryo-TEM images were not obtained after thin sectioning; therefore, it is not unexpected to observe gold nanoparticles that seemingly appear inside the EVs as a result of the tridimensionality of the object represented in 2D imaging.

23. *It is unclear from the study and discussion, which type of EVs (exosomes or plasma membrane derived EVs) the authors suggest to be of relevance for their findings. If I understand correctly, the authors argue that exosome-fractions also carry functional mitochondria. Have the authors tested whether the exosome fraction carry similar activity as crude EV-fractions (mito-EVs) regarding uptake and restoring mitochondrial functions in target cells (macrophages)? This would be a relevant addition to the manuscript.*

R23. While our paper mostly focuses on crude EVs, we agree that the role of the exosomal fraction was worth further investigation. As such, we have addressed the reviewer's questions with two completely new experiments.
First, we show that the fraction of macrophages incorporating MitoDSred particles via FACS analysis is significantly lower if these cells are treated with exosomes instead of crude EVs (**revised Figure 6b**). Second, we show that LPS stimulated macrophages treated with exosomes did not have any significant changes to either their metabolism or gene expression profile (**new Figure S4**).
These findings suggest that mitochondria are predominantly extruded by EVs from NSCs, albeit we cannot completely exclude technical issues related to the exosome purification protocol that might affect extracellular mitochondria functionality. We have discussed these findings (and limitations) in the main text and discussion in red.

24. *Fig. 9 c+d are not conclusive and difficult to interpret. How were MitoDsRed particles exactly determined? The labelling of the y-axis in 9c is not clear. Confocal Localization of particles often overlap with nuclei (GFAP and NeuN stain). The appearance of the fGFP in the images should be explained (do the transplanted NSCs form such long processes?). It would actually be interesting to compare control mice that did not receive immunization.*

R24. We have added further methodological details and descriptions of the pathological evidence presented (including details on areas sampled, z-stack projections, and NSC processes). In addition, we have performed a completely new transplantation experiment in mice that did not receive immunization, which were transplanted with fGFP$^+$MitoDSred$^+$ NSCs, as suggested by the reviewer. The quantification of the mitochondrial transfer events in these non-immunised mice has been compared to that of EAE mice, and it is now reported in the **revised Figure 9c**. Changes in the text and figure legends are in red.

25. *When screening TabS1 for EV-and Exo-enriched proteins, a number of proteins are turning up that are rather associated with differentiated neural cells (e.g. synaptic proteins and Proteolipid protein 1). How does this come about? Are the NSCs partly differentiated?*

R25. Our protocol for EV isolation entails the culturing overnight in a serum-free chemically defined (stem cell) proliferation medium that is supplemented with growth factors (EGF and FGF) that maintain their stemness, as described [4]. NSCs under these conditions are healthy,

with no changes in viability or self-renewal compared to NSCs cultured in normal conditions. We have provided further information on the media composition in the methods section in red.

26. *Fig. 1c and Fig. 3b: the dashed line actually indicates q<0.1 not 0.05*

R26. We thank the reviewer for noticing this. We have corrected the line height to reflect the value of q<0.05.

27. *Fig. 1f, WB: why do EV markers Pdcd6ip and Tsg101 appear prominently in Mito fraction?*

R27. The preparation of the Mito fraction was performed to obtain a mitochondria enriched fraction from cellular lysates. Therefore, it is possible that proteins such as Pdcd6ip and Tsg101 are present in this preparation, which is not pure by definition. We have made sure that this is explicitly mentioned in the text in red.

28. *Methods: please add details regarding Accumax.*

R28. We have added further information on Accumax in the methods section.

29. *Fig. 5b, figure legend is unclear: how was quantified? I understood the percentage of cells taking up mito-EVs is depicted. Then, what does n=9 cells per replicate mean?*

R29. We thank the reviewer for pointing out this incongruency. We have now better explained the methods of quantification and the number of replicates in the methods section and figure legend, as suggested.

30. *Fig. 6b, please label X-Axis of flow cytometry blots.*

R30. We have revised figure 6b, as suggested. It now includes a completely new set of experiments and a better depiction of the data.

31. *For the assays shown in Fig. 6+7, it would be interesting to see Mito in comparison to Mito-EVs. If the authors have the data, I would ask to share them with the community.*

R31. Since our data show that EVs are significantly more efficient than a mitochondria enriched fraction in increasing the survival of Rho0 cells (Figure 5), all the following experiments have been performed with EVs only (including those of figure 6 and 7). This has helped to focus the investigation, to provide all the necessary controls, and to identify a putative new therapeutic tool. Nonetheless, in order to address this reviewer's comment, we have now included in the discussion evidence coming from previously published data suggesting that mitochondria treatment alone is indeed a pro-inflammatory stimulus in leukocytes [6].

32. *Fig. 9c,d: please specify analysed/depicted CNS region in the legend.*

R32. We have adjusted the legend, as suggested.

33. *Please specify nature of error bars in legends.*

R33. We have adjusted the methods and legends to better specify the nature of the error bars, as suggested.

References

1 Indira, D. *et al.* Strategies for imaging mitophagy in high-resolution and high-throughput. *Eur J Cell Biol* **97**, 1-14, doi:10.1016/j.ejcb.2017.10.003 (2018).

2 Mitra, K. & Lippincott-Schwartz, J. Analysis of mitochondrial dynamics and functions using imaging approaches. *Curr Protoc Cell Biol* **Chapter 4**, Unit 4 25 21-21, doi:10.1002/0471143030.cb0425s46 (2010).

3 Schneider, A. *et al.* Single organelle analysis to characterize mitochondrial function and crosstalk during viral infection. *Sci Rep* **9**, 8492, doi:10.1038/s41598-019-44922-9 (2019).

4 Cossetti, C. *et al.* Extracellular vesicles from neural stem cells transfer IFN-gamma via Ifngr1 to activate Stat1 signaling in target cells. *Molecular cell* **56**, 193-204, doi:10.1016/j.molcel.2014.08.020 (2014).

5 Iraci, N. *et al.* Extracellular vesicles are independent metabolic units with asparaginase activity. *Nat Chem Biol* **13**, 951-955, doi:10.1038/nchembio.2422 (2017).

6 Unuma, K., Aki, T., Funakoshi, T., Hashimoto, K. & Uemura, K. Extrusion of mitochondrial contents from lipopolysaccharide-stimulated cells: Involvement of autophagy. *Autophagy* **11**, 1520-1536, doi:10.1080/15548627.2015.1063765 (2015).
