# Supplementary material for: Neural stem cells traffic functional mitochondria via extracellular vesicles
Source: PLoS Biol. 2021 Apr 7;19(4):e3001166. doi: 10.1371/journal.pbio.3001166 (PMC8055036; doi:10.1371/journal.pbio.3001166)
Supplement: Attachment — Submitted filename: PBIOLOGY-D-20-00273R3_Point by point reply.pdf [file pbio.3001166.s010.pdf]

Reviewer #1

1. *The authors have extensively addressed my concerns.*

R1. We thank this reviewer for acknowledging the extensive additional data and experiments that we managed to provide within this review. We now hope that our manuscript will be suitable for publication.

Academic Editor

2. *Based on the reviews, we will probably accept this manuscript for publication, provided you will clarify the lack of actin or a different loading control in Figure S1b.*

R2. We thank the Academic Editor for reviewing our manuscript. Regarding Figure S1b, the absence of beta-actin from the last two lanes is readily explainable. These two control lanes were requested by reviewer number 2, who was afraid that the commercial media in which we grow Neural Stem Cells (NSCs) and extract Extracellular Vesicles (EVs) might contain a low amount of EV contamination prior to cell culturing. With Figure S1b, we demonstrate that despite proteins were detected by BCA after ultracentrifugation of our modified commercially available media (see figure below), the presence of EV contaminants (as well as other proteins, such as beta-actin) was negligible (as shown in Figure S1b). We hope the Academic Editor will find this explanation sensible.

	Replicate 1	Replicate 2	Average	Corrected	Interpolated Values (µg/mL)	Dilution Adjusted (µg/mL)
**Samples:**						
CD63+	0.5413	0.5333	0.537	0.444	342.7009783	3427.01
CD63-	0.3045	0.3236	0.314	0.220	165.5156928	1655.16
NSCs	0.373	0.3645	0.369	0.275	208.1863717	2081.86
EVs	0.4467	0.4689	0.458	0.364	278.3755327	2783.76
NPC media	0.1094	0.1127	0.111	0.017	12.5035017	125.04
MV Media	0.1106	0.1155	0.113	0.019	13.97758504	139.78

Reviewer #3

3. *The authors comprehensively addressed all reviewer comments and added several new convincing experimental data. The manuscript has significantly improved, and all my concerns are fully satisfied.*

R3. We thank this reviewer for her support and for her guidance in improving the manuscript, which we hope will be now suitable for publication in PLOS Biology.

Ethics Statement

4. *Please include within your manuscript the full name of the IACUC/ethics committee that reviewed and approved the animal care and use protocol/permit/project license. Please also include an approval number. Please include the specific national or international regulations/guidelines to which your animal care and use protocol adhered. Please note that institutional or accreditation organization guidelines (such as AAALAC) do not meet this requirement.*

R4. The requested information has been provided in the materials and methods section (page 38, lines 1328-1332).

Data Policy

5. *Please provide the dataset identifier for http://proteomecentral.proteomexchange.org. Please update your Data Availability statement to include such identifier.*

R5. The proteomic dataset identifier has been provided in the materials and methods section. The data set will be made publicly available once we will have a DOI for the publication so the two are linked in the database. Until then then the Editors can access the data using the reviewer account with user name "reviewer_pxd024368@ebi.ac.uk" and password "x0rxCjzz". Changes in the text are reported in page 22, lines 753-754.

6. *In addition to the microarray and proteomic data, we need you to provide as the individual quantitative observations that underlie the data summarized in the figures and results of your paper. Regardless of the method selected, please ensure that you provide the individual numerical values that underlie the summary data displayed in the following figure panels: Figures 1bcd, 3bc, 4abefg, 5bc, 6bdef, 7c, 8acde, 9bc, S1a, S2a, and S4ab.*

R6. We have reviewed the data and statistical analysis of Figures 1bcd, 3bc, 4abefg, 5bc, 6bdef, 7c, 8acde, 9bc, S1a, S2a, and S4ab, and we are now providing supportive quantitative data in a new supplemental Excel spreadsheet (Data S3), as requested.

Blot and gel reporting

7. *For manuscripts submitted on or after 1st July 2019, we require the original, uncropped and minimally adjusted images supporting all blot and gel results reported in an article's figures or Supporting Information files. We will require these files before a manuscript can be accepted so please prepare and upload them now. Please carefully read our guidelines for how to prepare and upload this data: https://journals.plos.org/plosbiology/s/figures#loc-blot-and-gel-reporting-requirements.*

R7. The requested uncropped gels have been provided.
